# Haemophilia A: A Review of Clinical Manifestations, Treatment, Mutations, and the Development of Inhibitors

**DOI:** 10.3390/hematolrep15010014

**Published:** 2023-02-16

**Authors:** Samuel Sarmiento Doncel, Gina Alejandra Díaz Mosquera, Javier Mauricio Cortes, Carol Agudelo Rico, Francisco Javier Meza Cadavid, Ronald Guillermo Peláez

**Affiliations:** 1 Integral Solutions SD SAS, Integral Solutions Research, Bogota 110121, Colombia; 2 Life Sciences and Health Research Group, Graduates School, CES University, Medellin 050021, Colombia; 3 Hospital Universitario San Jorge, Pereira 660002, Colombia

**Keywords:** Haemophilia A, inhibitors, mutations, treatment

## Abstract

The purpose of this narrative review was to provide an overview that allows readers to improve their understanding of hemophilia A, which is considered a genetic disease with a high impact on the quality of life of people who suffer from it is considered one of the diseases with the highest cost for health systems (In Colombia it is part of the five diseases with the greatest economic impact). After this exhaustive review, we can see that the treatment of hemophilia is on the way to precision medicine, which involves genetic variables specific to each race and ethnicity, pharmacokinetics (PK), as well as environmental factors and lifestyle. Knowing the impact of each of these variables and their relationship with the efficacy of treatment (prophylaxis: regular infusion of the missing clotting factor VIII in order to prevent spontaneous bleeding) will allow for individualizing the medical behavior in a cost-effective way. For this is required to build more strong scientific evidence with statistical power that allows us to infer.

## 1. Methods

For this documentary research, publications were reviewed that considered knowledge about haemophilia A, its response to treatment, immunogenicity, bleeding episodes, quality of life, pharmacokinetics, treatment, and the development of inhibitors.

The search was performed according to the following terms: haemophilia A, coagulation disorder, coagulation factor disorder, coagulation factor deficiency, and coagulation factor disease.

## 2. Physiology of the Blood Coagulation System

### 2.1. Haemostasis

Haemostasis is the process by which haemorrhagic processes are controlled. This process includes the interaction between different blood components and the vessel wall, which blocks the outflow of blood at the site of tissue damage [1]. Initially, it was thought that haemostasis was activated by two pathways that converge in a common pathway, the intrinsic pathway being the one that is activated from the components present in the blood, within which the coagulation factors are only some of the proteins that help haemostasis. When a blood vessel is injured, the walls contract to decrease blood flow to the area of injury, and platelets bind to the site of the injury to stop bleeding [2]. On the surface of the platelets, several coagulation factors work together in chemical reactions, also called the coagulation cascade, whose function is to form a fibrin clot [2,3].

Generally, these coagulation factors circulate in the blood in an inactive manner. When an injury is generated, each coagulation factor is activated in a specific order [2].

The extrinsic pathway, unlike the intrinsic pathway, requires a component external to the blood, such as a tissue factor (TF), or tissue thromboplastin, to initiate activation [2,3].

#### 2.1.1. Classical Theory 

In 1905, Morawitz presented the coagulation model based on four factors. It begins with prothrombin (factor II), which is activated through two factors (tissue factor and calcium) to produce thrombin, responsible for the conversion of fibrinogen to fibrin. In the 1950s, coagulation factors were found to be activated by a linear sequence of events or reactions from proteins that circulate in an inactive form as proenzymes. Each factor has a cofactor capable of activating another factor (V, VII, VIII, IX, XI, VWF). If there is an interruption or lack of some of them “in the coagulation cascade,” a haemostasis deficit is generated [1].

**Figure 1:**
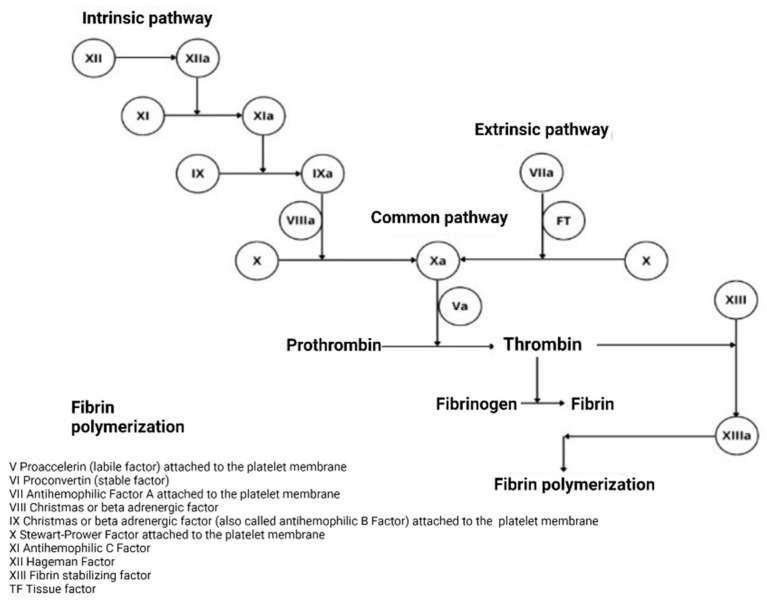
Classical concept of the coagulation cascade. Note: This figure presents the classical theory of coagulation based on two pathways (intrinsic and extrinsic) that converge in a common pathway. Mann KG, et al. [4].

Two pathways in the coagulation cascade were considered: the intrinsic pathway—factors present in the blood—and the extrinsic pathway, in which an external tissue factor (TF) was required to initiate activation. These converge in the common pathway after activation of the FXa. The main factor in the generation of factor IIa (Prothrombin) is the FXa factor, which is generated in the two pathways called tenses (FX enzyme). The presence of the cofactors FVIIIa and FVa is essential, as can be seen in Figure 1 [2,4].

The sequence of the classical theory based on the activation of coagulation factors is correct in this sense; however, the concept of two separate activation pathways that converge in a common pathway is wrong [2]. 

#### 2.1.2. The Cellular Model of Thrombin Formation

**Figure 2:**
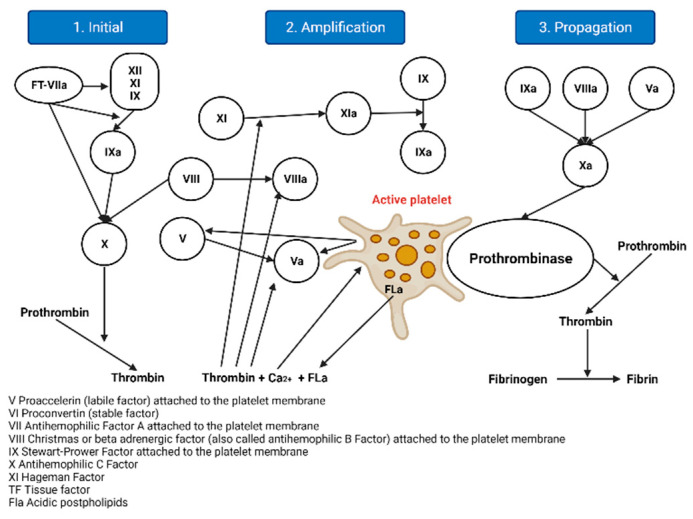
Current molecular model of coagulation. Note: This figure presents the current model of the coagulation cascade based on stages, created by the main author using the BioRender tool. Adapted from Sarmiento Doncel, S, et al.

This model is an advance since it does not consider two activation pathways that converge in a common pathway, but considers a single stepped or sequential process and proposes three stages, as evidenced in Figure 2 [2].

- Initiation: Haemostasis is initiated by vascular rupture, giving rise to the exposure of the tissue factor (TF), which is not usually in contact with blood. When this union with FVIIa occurs, a tenase complex is formed that activates FX by joining FV, forming a new complex, enzyme prothrombinase, which generates thrombin. In addition to activating FV, it is also activated by FXI, which results in FXa and thrombin, but in minimal amounts, since it is inhibited by the thrombin-inhibiting factor [5]. The extrinsic pathway is now the initiating physiological pathway that produces small amounts of thrombin and activates platelets, as mentioned above [2].
- Amplification: This stage occurs through positive feedback on the intrinsic and common pathways, generating large amounts of thrombin. It is known as amplification [2], as described below. The reduced amounts of thrombin (FIIa) formed in the initiation phase activate the FV cofactors, FXI, FXIII, and platelets, through protease-activated receptors; FVIII is separated from VWF by thrombin and the other factors are activated for the next phase [6,7].
- Spread: At this stage, the phase of fibrinogenesis and platelet aggregation begins [2]. It occurs on the surfaces of previously activated platelets, leaving receptors exposed for the binding of activated coagulation factors. It binds FXI, activating more FIX molecules by binding to its cofactor FVIII and forming a more potent defence complex, forming more FXa. FXA binds the FV cofactor again, which leads to the generation of more thrombin, but it is no longer inhibited [6,7].

## 3. Haemophilia A

Haemophilia A is a hereditary disease generated by a genetic defect in the long arm of the X chromosome, causing a qualitative or quantitative deficiency of coagulation factor VIII in the case of haemophilia A [8,9,10]. 

It is important to clarify that haemophilia only has two types: A and B. However, there are two coagulation disorders that used to be confused with haemophilia: factor XI deficiency and von Willebrand disease (vW). It was thought that the deficiency of factor XI was haemophilia since it is part of the intrinsic pathway, similar to factors VIII and IX; however, factor XI deficiency is an autosomal hereditary disease and is not linked to sex chromosomes and occurs in the same proportion for both men and women; the bleeding outcomes are also different and are considered less severe. On the other hand, vW disease is a more incident and prevalent coagulation disorder, but it is not a form of haemophilia and is caused by a deficiency of vWV factor, which is a protein that helps factor VIII as a chaperone protein, preventing it from being destroyed by nucleases, and acting as a platelet activator.

Regarding the above, the most frequent type of haemophilia is haemophilia A, representing 80 to 85% of haemophiliacs. Haemophilia B represents approximately 15% [11]. 

### 3.1. History

The first reported cases of haemophilia A date back to Ancient Egypt, according to writings found in ancient papyri. Others date back to reports of Hebrew texts (Talmud) from the 2nd century AD [12]. These texts show that, at that time, there was a hereditary relationship in patients with haemostasis problems, and they decreed that in those children with a family history of haemorrhage after circumcision, this procedure was prohibited [12]. By the 19th century, this disease was defined as the disease of kings, since Queen Victoria of England transmitted the disease to her descendants, and these in turn to several of the royal houses of Europe and Russia [12]. 

### 3.2. Genetic Etiology

Haemophilia A is an inherited disease caused by a defect in the gene located on the long arm of the X chromosome, causing a qualitative or quantitative deficiency of coagulation factor VIII [8,9,10]. Inheritance of haemophilia A is linked to (sex) chromosome X; therefore, men who have a defective copy of the FVIII gene on their X chromosome will pass on a normal Y chromosome to all their male children and an abnormal X chromosome to all their female daughters: their sons will be unaffected, and all of their female daughters will be carriers [13]. For female carriers, at each birth, there is a 50% chance of transmitting the disorder to their sons and a 50% chance that female daughters will be carriers, except in rare situations of homozygosis or double heterozygosis [9,10,11,13]. 

Up to a third of haemophilia cases are sporadic, presenting through spontaneous mutations, which means that there is no evidence of them in family history [9].

### 3.3. Epidemiology

Haemophilia A is estimated to affect 1 in 5000 to 10,000 male live births [12,14]. According to the report published in 2011 by the World Federation of Haemophilia (WFH), Colombia has a prevalence of 5.2 per 100,000 men [9]. For the year 2019, the reported high-cost account (HAC) was 1916 patients with haemophilia A [15]. 

### 3.4. Clinical Features

Haemophilia is characterised by a deficiency of coagulation factors that leads to a decrease in haemostasis [8], which generates the presence of spontaneous bleeding that varies in frequency and severity depending on the level of factor present at the plasma level [9,10,12,16]:

▪ Mild deficiency (5–40% FVIII activity): It usually only presents with bleeding after surgical procedures.
▪ Moderate deficiency (1 to 5% FVIII activity).
▪ Severe deficiency (<1% FVIII activity): It is characterised by the presence of spontaneous bleeding more frequently, which leads to progressive damage mainly at the joint level [10,17]. It exhibits a severe bleeding phenotype and can present from the beginning of life [18].

#### Bleeding in FVIII Deficiency

Hemarthrosis: In younger children, it occurs in a greater proportion in the ankle than in the knees, and in older children mainly in the knees, rather than in the elbows, as in adults, with bruises, frenulum-type bleeding in the mucosa, epistaxis, prolonged bleeding due to trauma or surgeries, and, less frequently, hematuria, gastrointestinal bleeding, bleeding in the respiratory tract, and bleeding in the central nervous system [11].

The severity of the disease is related to the level of FVIII activity. For severe disease, the functional activity of the factor is below 1%. Joint bleeding leads to arthropathy, haemorrhagic shock, neurocognitive defects, and even death [11]. Haemophilia is a chronic disease that, without adequate treatment, generates disability, which impacts the quality of life of patients who suffer from it [19]. Approximately 70–80% of bleeding episodes affect the joints and cause hemarthrosis and haemophilic arthropathy [12].

Hemarthrosis mainly affects large joints such as the shoulders, elbows, ankles, knees, and hips. When recurrent bleeding occurs in the same joint, atrophic changes are generated and it is known as haemophilic arthropathy [20], whose course is chronic and disabling [9].

The frequency of bleeding events depends on their location [9,11]:

- Hemarthroses (70–80% incidence): the most common events occur in the knee, ankle, and elbow joints, and less frequently in the shoulders, wrists, and hips.
- Muscles: there is an incidence of 70–80%.
- Other important haemorrhages occur with an incidence of 5–10%.
- Central nervous system: there is an incidence of <5%. It is the main cause of mortality in severe haemophilia patients, but, fortunately, its incidence has been declining with the widespread introduction of prophylaxis. All head injuries that are accompanied by headache, drowsiness, or vomiting should be considered as possible intracranial bleeding and treated immediately. If the severe pain is at the level of the back, it may be a symptom of bleeding at the level of the spinal cord.

### 3.5. Description of Factor VIII

**Figure 3:**
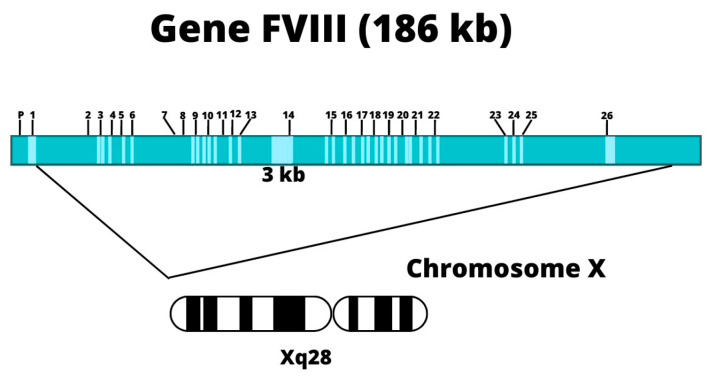
Structure of the FVIII gene. Note: Location of coagulation factor VIII on the X chromosome, taken from the Molecular Basis of Haemophilia [23]. Adapted from Sarmiento Doncel, S, et al. using the BioRender tool.

FVIII is a high-molecular-weight glycoprotein that acts as a cofactor in the coagulation cascade [9]. It is located on the long arm of the X chromosome at position Xq28 [10,14]; see Figure 3. The gene covers 186 Kb of genomic DNA, consists of 26 exons, and encodes a 9.0 Kb [21] messenger RNA (mRNA), encoding a precursor polypeptide of 2351 amino acids, synthesised mainly by liver cells (hepatocytes) [10,14,22].

**Figure 4:**
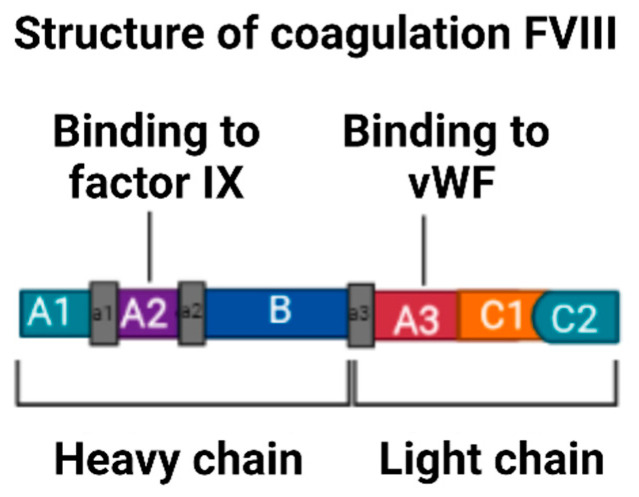
Structure of coagulation FVIII. Note: This figure shows the structure of FVIII. After thrombin activation, this protein becomes a heterodimer with a heavy chain (A1-A2-B) and a light chain (A3-C1-C2). Figure created by the main author using the BioRender tool. Adapted from Hernandez C, et al. [25].

The mature protein of FVIII is composed of a heterodimer with two chains composed of three homologous A domains, a single B domain, and two homologous C domains that are in the order (A1-A2-B) heavy chain and (A3-C1-C2) light chain [24], from the amino terminus to the carboxyl terminus [14], as is evident in Figure 4. The domains are of vital importance in the function of FVIII because each one contains specific binding sites for components of the coagulation cascade [14].

FVIII is an unstable factor that circulates in plasma, bound and transported by the von Willebrand factor (vWF), which acts as a chaperone molecule [10]. It has a half-life of approximately 12 h in adults, and, in children, it tends to be shorter [10]. Factor VIII is protected from proteolytic degradation by VWF and concentrates at sites where a vascular injury is present [10]. The FVIII protein is necessary for the propagation of the coagulation cascade and thus achieving haemostasis [14].

### 3.6. Inhibitors in Haemophilia

An inhibitor is an immunoglobulin G (IgG) type antibody, and its function is to destroy substances that it does not recognise as its own. Approximately 30% of patients with severe HA develop antibodies against FVIII (also called “inhibitors”) due to an immunological response to treatment as an adverse event of replacement therapy [26,27].

These inhibitors (antibodies) are a way in which the immune system generates protection against external agents that it detects as foreign [27]. When people with haemophilia are exposed to exogenous coagulation FVIII, the immune system perceives the treatment as a foreign protein in the body and develops neutralising alloantibodies (antibodies that are generated as a result of exposure to a foreign organism) against the supplied FVIII [27].

Once the inhibitors directed against the exogenous coagulation of FVIII are generated, it is neutralised depending on the number of antibodies generated, measured in Bethesda units. This inhibition also occurs against the synthesised endogenous factor, but to a lesser extent [28]. The presence of these inhibitors increases mortality and morbidity since the patient does not respond to standard therapy, making treatment ineffective [27,28].

There are two types of inhibitors: low-response, when their level is maintained at ≤5 Bethesda units per ml (BU/mL), and high-response inhibitors, when their level is ≥5 BU/mL [28]. One Bethesda unit is the concentration capable of inhibiting half of FVIII in a mixture of patient plasma and normal plasma [28].

### 3.7. Pathophysiology of the Inmune Response to FVIII

The cellular cascade begins with the contact of FVIII and APCs, which generates the endocytosis of FVIII through vesicles, generating the union of peptide sequences of FVIII with molecules of the major histocompatibility complex type II (MHC II) [29].

**Figure 5:**
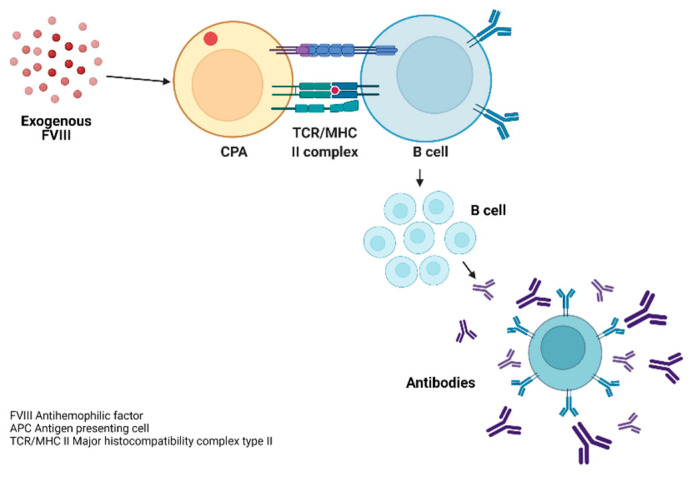
Development of inhibitors against exogenous FVIII. Note: Development of inhibitors against exogenous FVIII in patients with severe haemophilia A. Figure created by the main author using the BioRender tool.

The recognition of the complex (FVIII and MHC II antigens) occurs through the T-lymphocyte receptor (TCR) and CD45. This binding of the receptor to the antigen (Ag) is not generated in an isolated manner since it requires co-stimulatory pathways (ligand complex), the receptors necessary for lymphocyte activation and regulation. From these interactions of lymphocyte subpopulations, the production of antibodies against FVIII is generated through the final differentiation of B lymphocytes into plasma cells, which secrete large amounts of the antibody, as shown in Figure 5. These generated antibodies are generally IgG4 polyclonal and, in some cases, IgG1 [29].

### 3.8. Development of Inhibitors in Haemophilia A

The development of inhibitors is the most important complication during treatment with coagulation factors in patients with haemophilia A. It occurs in 20% to 30% of patients with severe haemophilia A [30,31] and generates an increase in bleeding episodes [8,30] since it inhibits its pro-coagulant activity.

Replacement therapy induces the development of alloantibodies, as the recognition of the exogenous protein as an antigen neutralises the haemostatic effect of FVIII, cancelling out the benefit of treatment. These IgG4 subclass antibodies usually appear within the first 25 repeated antigenic exposures to factor concentrates VIII [32,33]. The Bethesda assay (explained in the Diagnostic Tests section) is the gold standard for measuring FVIII inhibitor titres [26]. The severity of factor VIII inactivation depends on the inhibitor titres present, being higher for high-response inhibitors >5 Bethesda units (BU) than for low-response inhibitors <5 BU [16]. The eradication of inhibitors by immune tolerance induction (ITI) is successful in approximately two thirds of cases. This is a strategy whose objective is to decrease the immune response to the presence of FVIII in haemophilia A through frequent and intensive exposure to coagulation factor VIII [16].

Inhibitor titres are divided into [27] the following:

▪ Negative inhibitor titre: The titre is below the limit of detection of inhibitors in the local laboratory. If the reference value of the local laboratory is not available, any titre <0.6 Bethesda unit (BU) will be considered negative [27].
▪ A low titre of inhibitor: Any titre that is between the limit of detection of inhibitors of the local laboratory and ≤5 BU will be considered a low-titre inhibitor. If the local laboratory reference value is not available, any titre ≥0.6 BU will be considered a low-titre inhibitor [27].
▪ A high titre of inhibitor: Any titre ≥5 BU at any time after diagnosis will be considered a high-titre inhibitor [27].

#### 3.8.1. Incidence of Inhibitors in a Previously Treated Patient (PTP) in Haemophilia

Previously treated patients are defined as those who have reached 150 days of exposure to coagulation factor VIII [27]. The highest risk for the development of inhibitors is stipulated during the first 20 to 50 exposures to exogenous coagulation factor FVIII: after this period, the risk decreases considerably. The incidence of inhibitors in PTP is 2–5 per 1000 treated patients [34].

#### 3.8.2. Incidence of Inhibitors in Treatment-Naive Patients (PUP)

PUP patients are defined as those patients not previously treated and are grouped from day one of exposure to coagulation FVIII to day 50 of exposure. In a study conducted with more than 600 patients, the majority with a diagnosis of SHA who had not received prior treatment, the presence of inhibitors occurred after a median of 15 SD [27,34]. The cumulative incidence of inhibitors in PUPs with severe haemophilia A was 25.9% [34].

### 3.9. Risk Factors Associated with the Development of Inhibitors

Several factors associated with the generation of inhibitors have been described, among which are the type of genetic mutation, family history, race, major histocompatibility complex (HLA) class I/II, severity of haemophilia, and genetic polymorphisms in interleukin-10 (IL-10) and tissue necrosis factor, as well as factors related to treatment, such as the type of factor concentrate (plasma, recombinant), the frequency of treatment, as well as the age at the initiation of prophylaxis [27,30,33,35,36], as shown in Table 1. Within the non-modifiable factors related to the patient, we find functional factor VIII and genetic mutation [37]; this is how those mutations that present large deletions, nonsense mutations, and intron-22 inversion present a high prevalence of inhibitors, being 7 ± 10 times higher than in patients with nonsense mutations, small deletions, and mutations in the intron-22 splice site [30].

In a systematic review and meta-analysis of the type of mutation of the FVIII gene and the development of inhibitors in patients with severe haemophilia A, 30 studies were included that comprised 5383 patients, of whom 1029 had inhibitors. As a result, it was found that mutations such as large nonsense deletions and mutations presented a high risk of inhibitors (OR = 3.6 95% CI 2.3–5.7, and OR = 1.4 95% CI 1.1–1.8, respectively) [38]. and a low risk for small deletions or insertions and missense mutations, with OR = 0.5 and 95% CI 0.4–0.6, and OR = 0.3, CI 95% of 0.2–0.4, respectively [38]. In this study, it was possible to conclude that the FVIII genotype is an important determinant for the development of inhibitors in patients with severe haemophilia [38].

#### 3.9.1. Immune Response in Haemophilia

Currently, the immune response remains a mystery because some patients with haemophilia have inhibitors and others do not, although it is known that some patients are at increased risk due to a combination of genetic and environmental factors [27,39].

#### 3.9.2. Major Histocompatibility Complex in Haemophilia

The MHC is associated with the formation of inhibitors, as described by J. Oldenburg et al. Each MHC recognises a unique peptide of nine amino acids, which corresponds to an HLA allele. HLA class I/II alleles and the tumour necrosis factor α (TNFA) locus are closely linked in the MHC complex [32,39,40,41]. 

Studies by Oldenburg et al. [40] and Hay et al. [41] showed that some HLA class II alleles are associated with a high risk of inhibitor formation. However, some alleles have been shown to prevent the generation of inhibitors. Both studies included patients with intron-22 inversion; some of these patients generated inhibitors, while others did not. The results obtained from HLA-II allele identification showed that the most frequent alleles in patients with haemophilia that generated inhibitors are DR15 and DQB0602, with a relative risk of 2.2 and 3.7, respectively. Therefore, these alleles were classified as “risk” alleles, while DR13 and DQB0603 were classified as “protective” [32,40]. Meanwhile, Hay et al. found similar results with respect to the frequency of these alleles and the formation of inhibitors [41]. However, Astermark et al. found no consistent association among these findings [32].

#### 3.9.3. TNF-α

This is an important cytokine with pro-inflammatory and immunomodulatory functions, and it is associated with alloantibody production in patients with haemophilia [32].

#### 3.9.4. Race and Ethnicity

FVIII inhibitors have been found to be more common in African American (AA) and Hispanic (H) patients than in Caucasian (C) patients [42]. The Colombian population is characterised by miscegenation, mainly between indigenous natives, descendants of the Iberian Peninsula, and Black individuals from Africa [43]. This miscegenation poses a genetic pool that can vary in terms of the prevalence, phenotype, and severity of the disease.

According to a study by Carpenter SL et al., black people have a higher prevalence of inhibitors than white people. In a cross-sectional study of 5651 men with severe haemophilia A by Carpenter SL et al., the prevalence of inhibitors was compared in various racial and ethnic groups participating in the Universal Data Collection (UDC) project sponsored by the Centers for Disease Control and Disease Prevention. In this study, ethnicity was classified by distributing white as a dichotomous variable (Hispanic and non-Hispanic), and the same occurred for Black, Asian, and Native American populations, among others. As a result, the prevalence of inhibitors was found to be higher among Black and Hispanic individuals, at 26.8% and 24.5%, respectively, and lower among non-Hispanic white individuals (16.4%) (OR 1.4; CI 95%: 1.1, 1.7) [44]. 

### 3.10. Mutation Responsible for Haemophilia A

Genetic defects can affect the interacting domains (A1-A2-B-A3-C1-C2) and cause haemophilia A [14]. Since the 1984 publication of the FVIII gene sequence, a large number of mutations have been identified that can cause HA, the most common mutations being intron-22 (Inv22) and intron-1 (Inv1) inversion, affecting approximately 40–50% of the population with HA [10,22,24,45]. There are currently more than 1209 mutations within the coding and untranslated regions that are part of the HAMSTerRS mutation database (Haemophilia A Mutation Database and Factor VIII Resource Site), in which they report type mutations: point (substitution of a nucleotide), deletions, insertions, rearrangements, and inversions [22]. People with mutations such as intron-22 inversion, deletions, and nonsense mutations have severe haemophilia A, with a higher risk of generating inhibitors [46].

The bleeding phenotype (defined as the annual frequency of bleeding, including joint and non-joint bleeds, annual consumption of factor concentrates, and markers of joint status) correlates with the type of FVIII gene mutation and is also related to levels of residual FVIII factor in plasma. Depending on the type of genetic mutation that causes haemophilia A, some authors have classified having or not having FVIII residues or synthesis as non-null and null, respectively [47]. Null mutations include intron-22 inversion, which is responsible for severe haemophilia A [47].

Determining the FVIII mutation early in life and before starting treatment may allow a prediction of the bleeding pattern in newborn infants, help to better predict the risk of inhibitor development, and enable clinical decision-making [16,47].

Depending on the type of genetic mutation that causes haemophilia A, some authors have classified having or not having FVIII residues or synthesis as non-null and null, respectively. The changed ones in which there is no factor synthesis (null) present a more severe phenotype in the disease and the development of inhibitors [47].

**Intron-22 inversion (INV 22):** Intron-22 inversion is the most frequent mutation in SHA. Intron-22 is the largest, with a length of 40 Kb [48]. This inversion is generated by homologous recombination between the intragenic region (int22h1) and the extragenic regions (int22h2 and int22h3) [49] and a translocation of exons 1 to 22, which gives rise to the complete absence of expression of factor VIII [49,50,51].

**Intron-1 inversion:** It represents less than 5% of SHA cases in the Caucasian population [24]. It is generated by intrachromosomal recombination between a sequence (1 Kb) in intron 1 (int1h1) and a homologous sequence (inth1h2) separated by 140 Kb in the 5’ direction [48,51] in the telomere, resulting in a split FVIII gene, with the promoter and exon 1 separated from the remaining gene, resulting in severe haemophilia A [51].

**Deletions or insertions:** 

- Large deletions or insertions: These are those in which more than 50 bp [52] is added or lost, which alters the reading frame.
- Small deletions or insertions: These are defined by the loss of less than 50 base pairs (bp), generating changes in the reading (frameshift) and generating a premature stop codon [52]. This class of mutations is caused by polymerase slippage during the DNA replication phase [30]. Most patients with severe haemophilia A have large deletions and insertions [30].

**Point mutations:** 

- Mutations of amino acid change or missense:

The substitution of a nucleotide generates an amino acid change in the protein to be encoded (it can generate a mild, moderate, or severe phenotype, depending on the affected amino acid) [22]—for example, the Arg372His substitution of a thrombin cleavage site required for full activation of FVIII [53,54,55].

**Nonsense mutation:**

- This produces a stop codon, where a termination codon is produced (TAA, TAG, TGA). It is responsible for a severe phenotype [22].

#### 3.10.1. Background in the Literature on the Relationship between FVIII Mutations and Inhibitor Development

In the study conducted by Mannucci et al. in the years 2010 and 2014, 251 children with severe haemophilia A under 6 years of age, without inhibitors and minimally treated by less than five exposures to FVIII, from 42 centres in 14 countries (India, Egypt, Iran, United States Italy, and others) participated. This study predicted the development of factor VIII inhibitors in the SIPPET cohort [37], by mutational analysis and measurement of factor VIII antigen. These patients were randomised 1:1 to receive plasma or recombinant FVIII and were followed until the development of inhibitors. In the same way, the type of FVIII mutation was identified. Of these patients, 125 received plasma treatment, of which 29 patients (23.2%) generated inhibitors, and 20 of these patients (16.0%) generated high titres [37]. The other 126 patients received recombinant medication, of which 47 generated inhibitors (37.3%), and 30 of these (23.8%) generated high titres [37]. The other 126 patients received recombinant medication, of which 47 generated inhibitors (37.3%), and 30 of these (23.8%) generated high titres [37]. As a result, recombinant FVIII drugs were found to nearly double the rate of inhibitor development versus plasma-derived FVIII, with a hazard ratio of 1.87; 95% CI, 1.17 to 2.96; however, this estimate was not significant, which was probably due to the sample size and design [37]. Except for the type of mutation, no association was found between the variables analysed, such as the intensity of treatment, type of factor, ethnic group, and age at initiation of prophylaxis [37]. Based on this study by Mannucci et al., in 2018, a new study was conducted that sought to identify the mutations causing the generation of inhibitors. Of the initial 251 patients in the SIPPET study, 235 patients participated in this study. These patients came from 13 countries: India (*n* = 82), Egypt (*n* = 70), Iran (*n* = 30), United States (*n* = 16), Italy (*n* = 8), Mexico (*n* = 5), Spain (*n* = 5), Chile (*n* = 4), Austria (n = 4), Brazil (n = 4), Turkey (n = 3), Argentina (n = 2), and South Africa (*n* = 2). A causal variant was identified in 231 patients, of which 110 were intron-22 inversion mutations, 99 point mutations, 16 single- and multiple-exon deletions, and 6 with intron-1 inversion [16].

This study compared its mutation results from the SIPPET cohort with a meta-analysis that included 5383 patients with severe haemophilia A [38], showing a similar distribution: around half of the patients with intron-22 inversion and a higher frequency in nonsense mutations and lower frequency in missense mutations. Of the 231 patients, 72 presented a variant identified as causal and generated inhibitors, with a cumulative incidence of inhibitors of 36.3% (95% CI 29.4–43.2) [16]. A two-fold increase in inhibitor development was found for null mutations vs. non-null, with a hazard ratio (HR) of 2.08, 95% CI 0.84–5.17, and a 3.5-fold increase in inhibitor development for antigen-negative mutations compared with antigen-negative mutations with positive antigen (HR 3.61, 95% CI: 0.89–14.74) [16]. According to the results of this study, the association between the synthesis of minimal amounts of FVIII was confirmed, showing that, in these cases, the possibility of the development of inhibitors was reduced, and underlining the importance of investigating the levels of residual antigens of FVIII associated with causal variants to understand its clinical relevance [16].

According to the study by Mantilla et al. conducted in Mexican patients with severe haemophilia A in 2007, a frequency of intron-22 inversion (INV22) of 14/31 (45%) and 0% in intron-1 inversion (Inv1) [56] was presented. Inv22-positive patients showed a 1.88-fold increased risk of developing inhibitors; however, this OR value was not significant [56]. In 2010, a genetic analysis of HA was carried out in Taiwan in 115 patients that aimed to identify the most frequent mutations. These participants presented 79 severe, 15 moderate, and 21 mild phenotypes [57]. These reported data are clearly different from known data in the Caucasian population. Thirty-two samples (27.8%) were identified with Inv22 and six (5.2%) with Inv1. Twenty-one mutations were discovered that were not reported in HAMSTeRS. It was concluded that the prevalence of Inv22 in Chinese or Taiwanese patients with severe HA is 35% to 38%. Likewise, lnv22, Inv1, and large deletions are risk factors for the development of inhibitors. Sixty-five patients had missense mutations, nonsense mutations, insertions, and splice mutations [57]. In a study conducted in 2012 by Gouw SC et al. on the type of FVIII mutation and development of inhibitors in patients with SHA in a systematic review and meta-analysis, 30 studies were obtained as results for data analysis, where 5383 patients presented 3% large deletions, 10% nonsense mutations, 45% Inv22, 2% Inv1, 16% small deletions or insertions, 15% nonsense mutations, and 3% splice site mutations. In 4.6% of patients, the mutation was unknown [38]. By 2018, a new mutation in the FVIII gene was identified that generates severe HA. In a case study, a 68-year-old patient with factor activity of less than 0.001% never generated inhibitors. As a result, a new mutation causing severe HA was identified, which is a deletion identified as (c.5411_5413 del TCT, p.F1804 del) [58].

In a study by Albánez S et al., carried out on patients with severe haemophilia A in Venezuela, the aim was to identify the mutations of the FVIII gene in 50 patients. Identifying 49 mutations (91%) causing haemophilia, 41% presented Inv22. Inv1 was not detected in any of the study patients. For the remaining 27 patients, different mutations, deletions, insertions, and splice mutations, among others, were found. Seven new mutations were found, two of which were nonsense mutations, resulting in a premature stop codon in exons 11 and 17. Three were frameshifts, two caused by a base deletion, and one caused by an insertion (c.3006delG, c.6535delG, and c.5466–5471insA) that introduced a premature stop codon in exons 14, 23, and 16 [59].

#### 3.10.2. History of Variants or Mutations in Colombia

In the study carried out by Yunis et al. on a sample of 33 patients from the city of Bogotá, they sought to characterise, in a cost-effective manner, the identification of the mutation by inversion of intron 22 and intron 1. Finding a prevalence in the inversion of intron 22 of 42.4% (*n* = 14) and intron 1 inversion of 9.1% (*n* = 3), they also found that 20 samples presented Inv22, Inv1, or large deletions and 19 of them corresponded to severe haemophilia A. Eleven samples had point mutations or small deletions, and seven had severe haemophilia A. The rest of the samples presented missense variants and had a moderate or mild phenotype; on the other hand, two samples did not present genetic variation [46]. This study allowed us to conclude that a severe phenotype is mainly associated with Inv22, Inv1, large deletions, and frameshift. On the other hand, the nonsense and missense variants were associated with moderate and mild phenotypes [46].

In 2017, Garcés MF et al. conducted a study in Bogotá, where they sought to determine the frequency of Inv22 and Inv1 in 30 children with SHA, analysing only these two mutations [49]. The study showed that 12 patients (40%) presented Inv22 and three patients (10%) Inv1. It was concluded that the presence of inhibitors in the patients who presented INV22 was 41.6%, and in those who presented INV1, it was 33%. However, with INV1, the prevalence in the population of this study (10%) was higher than the frequency observed in other Latin American populations with 0%. This variation may be due to the number of patients evaluated, the family history of inhibitors, and ethnicity (Afro descendants), which are factors related to the development of inhibitory antibodies. In Colombia, no studies have been carried out in which the genetic mutations of coagulation FVIII and their association with the development of inhibitors have been evaluated [60].

In addition to the FVIII genotype, African American and Hispanic individuals in the United States have been found to be up to twice as likely to develop inhibitors [60]. Therefore, it is important to evaluate the clinical characteristics of Colombian patients with severe haemophilia A and their association with mutations and the generation of inhibitors.

### 3.11. Laboratory Diagnostic Test

#### 3.11.1. Methods That Measure the Functional Activity of FVIII

These methods are classified as coagulometric and chromogenic [61]. The coagulometry method measures the time it takes for a clot to form and is measured in the plasma using a sensor that captures changes in light. The chromogenic method measures the reaction by photometry (release of a coloured compound), as explained in detail below [61]:

#### 3.11.2. Chromogenic Method (Ccro) of FVIII

This method was developed in 1995 and is based on the fact that activated FVIII (FVIIIa) accelerates the conversion of factor X (FX) to activated FX (FXa) in the presence of activated factor IX, acidic phospholipids, and calcium ions [62,63]. The activity of activated FX (FXa) is evaluated by the hydrolysis of the chromogenic substrate p-nitro aniline specific for FXa [62,63] and the initial release rate of p-nitroaniline (coloured yellow). Therefore, the activity of FXa is proportional to the amount of functional FVIII present in the sample [62,63].

#### 3.11.3. Coagulometric or One-Stage Assay

This method is based on the measurement of the time it takes for a clot to form and from the activated partial thromboplastin time (TTPa), which is prolonged when there is a deficiency of FVIII, among others. Since the TTPa measures the time for the generation of fibrin, the patient’s plasma is taken together with a plasma sample with deficient FVIII, which are incubated with the reagent that contains a contact activator that can be kaolin or silica, among others, and phospholipids; calcium chloride is added to promote fibrin formation, measuring the time it takes to generate the clot, and this value is compared with a standard value [62,63].

#### 3.11.4. Quantitative Measurement of Factor VIII Inhibitors

Some patients with haemophilia A who are treated with exogenous FVIII may develop inhibitors. These inhibitors are quantified using the Bethesda test, which consists of mixing normal plasma (source of FVIII) with the patient’s plasma and incubating it for two hours at 37 °C, and then subjecting it to factor VII functional activity tests [64].

One unit of inhibitor (Bethesda unit or BU) is defined as the amount of inhibitor that will neutralise (destroy) 50% of one unit of factor VIII [64].

### 3.12. Treatment

The therapeutic approach aims to correct the factor deficiency through the administration of recombinant or plasmatic factor VIII concentrate [16]. This treatment has been developing new therapeutic strategies since the 1970s. On the one hand, there is conventional treatment (on demand), which consists of applying the treatment after presenting a bleeding episode [9,11,12,17] and whose objective is to achieve haemostasis in this event of bleeding [9]. It can also be used in those patients with mild or moderate haemophilia A who require a surgical procedure [9].

On the other hand, there is a treatment called prophylaxis. According to the World Health Organization (WHO), the World Federation of Haemophilia (WFH), and other scientific societies, prophylaxis is the first treatment option for severe haemophilia [9,11]. The main objective of prophylaxis is to increase the level of FVIII above 1 IU/dl to convert the bleeding phenotype from severe to moderate and to better preserve joint function [11,17], which allows a reduction in mortality and improves the quality of life of patients [16].

Prophylaxis (the frequent application of the factor) has been defined in three types, according to the start of treatment [9,11]:

▪ Primary: This begins before the second joint bleed, without the presence of joint damage, and prophylaxis treatment started before the age of three.
▪ Secondary: This begins after two or more joint bleeds, but before joint damage is established.
▪ Tertiary: This begins after joint damage has been confirmed by diagnostic means.

Among the most used protocols for prophylaxis in patients with haemophilia are the Malmo and Utrecht Protocols.

Under the Malmo Protocol, a 25 to 40 IU/Kg dose is given intravenously for three days each week in the case of SHA [11]. Under the Utrecht Protocol, a 15 to 30 IU/Kg dose is administered intravenously for three days each week in the case of SHA [11]. The Malmo Protocol is considered to be the gold standard [65].

The Dutch model individualises the dose and frequency of prophylaxis, adjusting it to clinical manifestations (recurrent spontaneous bleeding and joint status) without focusing on FVIII trough levels [66]. By focusing on phenotype rather than severity from the trough level of the factor, this model tends to be very cost-effective.

The Canadian model proposes staggered doses beginning with weekly infusions of factor VIII and, based on the phenotype or bleeding pattern, a gradual increase in dose is made (50 IU/kg/1 time a week, 30 IU/kg/2 times a week, and 25 IU/kg/3 times a week) [67]. This model requires fewer infusions.

Colombia, in its management guide, has accepted several protocols among these protocols, establishing it at 15 to 40 IU/Kg, a dose administered intravenously, three times a week, for patients with SHA [9].

Given the knowledge that trough levels above 1% of FVIII prevent spontaneous bleeding and therefore joint disease, in haemophilia A, 1 IU of FVIII modifies the factor concentration by 2%, which seeks to normalise haemostasis; the higher the dose, the higher the percentage of factor in the blood, which is why the concept of pharmacokinetics becomes a priority in the schemes of prophylaxis dosages defined as changes in concentration as a function of time [11]. Allowing an individualised dosage based on the absorption and elimination of each patient once it is administered, in this way, the use of coagulation factors is optimised [11].

#### 3.12.1. Pharmacokinetics (PK)

Pharmacokinetics (PK), as a field of pharmacology, is the one that studies the processes that a drug undergoes in the body, ranging from administration to absorption, distribution, metabolism, and elimination depending on the route of administration. In the case of FVIII, the administration is intravenous; therefore, the drug is 100% available from the time of administration [68].

Pharmacokinetics is being widely used in order to individualise treatments based on pharmacokinetic parameters in which the concentration of the factor is related to time, allowing the dose and frequency to be predicted according to each patient and the FVIII brand of coagulation to be administered, allowing improvements in effectiveness in relation to bleeding reduction and dose optimisation [11].

#### 3.12.2. Population Pharmacokinetics

From the development of Bayesian models whose statistical approach is based on the Bayes theorem developed in 1973 by Thomas Bayes, in which observations (events) are used to infer the probability of occurrences of a hypothesis, in this method, increasing the observations increases the possibility of the assertiveness of a hypothesis to be inferred. Thus, several tools are currently being used, including WhappsHemo and myPKFIT, which create individual pharmacokinetic profiles from the Bayesian model using pharmacokinetic data from population groups of patients divided by the use of coagulation factors with commercial brands, which allows one to lower the curve for the patient evaluated with said mark [69,70].

This pharmacokinetic model is currently a useful tool to establish the frequency and dose in patients with haemophilia [71]. Today, it is possible to calculate these pharmacokinetic curves from three samples, normally 3, 24, and 48 h after factor infusion, making the process much easier compared to the conventional pharmacokinetic study that requires a larger number of samples [71].

The study by Carlsson et al. evaluated standard prophylaxis (defined by the treating physician based on clinical and anthropometric variables such as weight) versus pharmacokinetic (PK)-guided prophylaxis, this being a randomised crossover study involving 21 patients, finding a reduction in factor consumption VIII of 32% with a similar bleeding rate in both scenarios over a 6-month period. In addition, PK-guided prophylaxis achieved better trough levels [71,72,73].

#### 3.12.3. Types of FVIII Products Used in Haemophilia A

##### Plasma-Derived Factor

These are factor replacement products that are created from human plasma. They are classified according to their degree of purity, such as intermediate-purity, high-purity, and ultrapure (UP), differentiated from each other by the different stages of viral inactivation [73].

For the year 1982, the Centers for Disease Control and Prevention (CDC) reported HIV infections presumably associated with administered FVIII factors [74], as well as HCV infections; by 1992, in the US, approximately 44% of patients with haemophilia had contracted HCV [12].

Today, plasma factors have evolved in their viral inactivation processes, being as safe as the factors developed by the recombinant technique [75,76].

##### Recombinant Factor VIII Concentrates

These are molecules manufactured after genetic manipulation of the host using recombinant DNA (rDNA) technology [75]. To this end, protein coding material is obtained from which a vector is constructed that must be adapted to the chosen host in order for the gene of interest to be expressed [77].

After the innovation in the development of recombinant factors, they have been evolving, seeking to reduce the frequency of infusions (longer times in the half-life), improving safety and efficacy. We may categorise into two large groups those with a standard half-life, which is characterised by having an average of 12 h of half-life [78], and those with an extended half-life (EHLF), which were developed in order to reduce the burden of treatment in prophylaxis, by reducing the number of weekly infusions of the factor, in turn maintaining adequate basal levels of the factor (concentration level above 1%) between infusion and infusion. Techniques used to prolong factor half-life include fusion (Fc receptor) and PEGylation technologies. These factors are widely used today, and no differential adverse events have been found vs. those with a standard half-life. In the case of EHL FVIII, it has been possible to increase the half-life between 1.4 and 1.6 times (approximately 7 h), reducing the number of infusions from three times a week to two times in most cases [78].

#### 3.12.4. Emicizumab

This medicine, authorised by the Food and Drug Administration (FDA) and the European Medicines Agency (EMA), is indicated for prophylaxis in all age groups in patients with haemophilia A and inhibitors. The recommended dose starts with the loading dose of 3 mg/kg once a week for the first 4 weeks, followed by a maintenance dose of 1.5 mg/kg once a week [79,80].

In recent years, several drugs have been developed based on mechanisms of action different from traditional treatment, such as Emicizumab, which is a recombinant human monoclonal antibody that bridges FIXa and FXa to restore the function of deficient FVIIIa in patients with haemophilia [79,81], necessary for effective haemostasis [80,81,82]. Due to its structure, Emicizumab is not expected to be affected by existing factor VIII inhibitors or to induce the new development of these inhibitors. It is subcutaneously given once a week and this even allows it to be spaced much further apart. This treatment has been shown to significantly reduce bleeding events in haemophilia A patients with and without inhibitors [83].

The efficacy and safety of Emicizumab have been evaluated in patients with and without inhibitors, where multicentre studies have been carried out in patients over 12 years of age with inhibitors (HAVEN-1) [84] and in the paediatric population (under 12 years of age) (HAVEN-2) [81]. In children and adults, they are weekly dose inhibitors (HAVEN-3) (HAVEN-3) [80,85]. These studies showed significant results in terms of efficacy in relation to the subcutaneous administration of the product and an infusion frequency of once a week, every two weeks, or even once a month, which has led to improved quality of life for patients. Similarly, a marked decrease in the annual bleeding rate (ABR) was observed, where it was reduced by more than 80% compared to on-demand treatment with FVIII and an increase in the number of patients with zero bleeding [81,84,85]. Subjects on Emicizumab prophylaxis had an ABR of 1.7 to 11.2 versus on-demand treatment with 12.3 to 43.9. This shows an advantage of prophylaxis with bypass agents and may offer the possibility of flexible treatment regimens according to the needs of the patient [86]. On the other hand, in the HAVEN-1 study, five serious adverse events were reported: one thrombophlebitis with skin necrosis, one cavernous sinus thrombosis, and three thrombotic microangiopathies (TMA) [84]. For these cases, the patients had been treated for bleeding concomitantly with Emicizumab and activated prothrombin complex concentrate (APCC) at doses greater than 100 IU/kg/day and for more than 24 h [81]. After the occurrence of these events, recommendations were established for the management of bleeding, such as using rFVIIa at the lowest dose (with a maximum initial dose of 90 μg/kg/d) and, if rFVIIa is not available, using a PCC at the lowest dose possible to control bleeding. In patients who have followed these recommendations, no cases of TMA or thrombosis have been reported [81,84,85].

#### 3.12.5. Treatment of Patients with Inhibitors

The treatment of bleeding in patients with inhibitors depends on BU titres. High responders should receive bypass agents when factor VIII is ineffective [87]. There are two referral agents, FEIBA (Activated Prothrombin Complex Concentrate) and Novoseven (Recombinant Activating Factor VII). These agents have different mechanisms of action but both have similar efficacy and an adequate safety profile. Bypassing agents can also be used in prophylaxis protocols in order to prevent the occurrence of bleeding [87]. Emicizumab is used for the prophylactic treatment of haemophilia A patients with inhibitors, but it is not used for the treatment of acute bleeding. Patients with low-titre inhibitors may receive factor VIII concentrates at higher than usual doses [87].

Induced immune tolerance (ITI) is a strategy whose objective is to reduce the immune response against the presence of FVIII in the case of haemophilia A through frequent and intensive exposure to coagulation factors (FVIII). Its success is around 75% in a period of up to three years. The patients who benefit the most from it are those with a high inhibitor titre (>5 BU) [88,89].

According to a meta-analysis carried out on 437 patients with data from the International Immunotolerance Registry (IRR, reported to an international registry) and the American Immunotolerance Registry (German and North American (NAIT) IT registries), it was found to be successful in 82% of cases when inhibitor values were less than 50 BU before starting ITI [88].

##### Immune Tolerance Regimens

Perhaps the most widely used regimen is the Bonn protocol, also called high-dose, which recommends the use of FVIII 100–150 IU/Kg every 12 h and can include the use of factor eight inhibitor bypass agent (FEIBA) or activated prothrombin complex concentrate (APCC) containing activated factors (particularly VIIa) at a dose of 50 IU/kg for 24 h [89,90,91].

The Dutch protocol suggests 25 IU/kg/24 h, and the Malmo protocol suggests 150 IU/kg every 12 h [88,89,90,91].

## Figures and Tables

**Table 1:** Non-modifiable and possibly modifiable risk factors for inhibitor development. Taken from Carcao M, et al. [27].

Risk Factors	Summary			Support Level
Non-Modifiable Genetic Risk Factors				
Type of FVIII mutation (null vs. non-null) and the position of the mutation	Type of mutation		Risk of inhibitor formation	Well established
	Null	Multidomain deletions	75%	
		Light-chain missense mutations	30–40%	
		Intron-22 inversion	20–25%	
		Domain deletions unique	15–25%	
		Small insertions/deletions in non-A zone	15–20%	
		Heavy-chain missense mutations	10–20%	
	Not null	FVIII missense mutations	<10%	
		Small insertions/deletions in zone A	<5%	
		Mutations at the splice site	<5%	
Family history	Risk 3.2 times higher (95% CI 2.1–4.9) if there was a family member with inhibitors			Well established
Ethnicity	1.9- to 4.7-fold increased risk in non-Caucasians (Black African > Latin American > Caucasian ancestry)			Established, but not well understood
TNF-α IL-10 CTLA-4 polymorphisms	TNF-α −308 A/A increases risk IL-10: allele 134 increases the risk CTLA-4: T-allele decreases risk			Some evidence, but not well understood
FVIII haplotypes	Haplotypes H3 or H4 have a higher risk of inhibitors since current FVIII products consist mainly of haplotypes H1 and H2			Reports discordant
Class I/II MHC genes or HLA polymorphisms	2-fold increased risk for HLADR15 and HLA-DQ6 and inhibitor formation			Reports discordant
Potentially Modifiable Environmental Risk Factors				
Trauma/surgeries	Major surgeries and trauma leading to treatment spikes increase the risk of inhibitor development			Established, but not well understood
Inflammation/infection	Could increase the formation of inhibitors			Established, but not well understood
Intense exposure, particularly at a young age	Increased risk of inhibitor formation			Established, but not well understood
Type of factor concentrate	Some studies suggest that conventional recombinant factor carries a higher risk of inhibitor formation than plasma-derived factor			Reports discordant
Early initiation of prophylaxis	Could confer true protection			No concrete evidence

## Data Availability

Not applicable.

## Abbreviations

Initials	Definition
aa	Amino acid
ADN	Deoxyribonucleic acid
ARN	Ribonucleic acid
EDTA	Ethylenediaminetetraacetic acid
FVIII	Factor VIII
FIX	Factor IX
FvW	Von Willebrand factor
HAMSTeRS	The Haemophilia A Mutation, Structure, Test and Resource Site
INV1	Intron-1 inversion
INV22	Intron-22 inversion
ITI	Immunetolerance
Kb	Kilobase
Pb	Base pair
PCR	Polymerase chain reaction
BU	Bethesda units
